# Inflammatory responses in epithelia: endotoxin-induced IL-6 secretion and iNOS/NO production are differentially regulated in mouse mammary epithelial cells

**DOI:** 10.1186/1476-9255-7-58

**Published:** 2010-11-30

**Authors:** Samar W Maalouf, Rabih S Talhouk, Floyd L Schanbacher

**Affiliations:** 1 Department of Animal Sciences, The Ohio State University, OARDC, Wooster, OH, USA; 2 Department of Biology, The American University of Beirut, Beirut, Lebanon; 3 IBSAR Nature Conservation Center for Sustainable Futures, The American University of Beirut, Beirut, Lebanon; 4 Department of Dairy and Animal Science, The Pennsylvania State University, University Park, PA, USA

## Abstract

**Background:**

IL-6 is a pro-inflammatory cytokine that signals via binding to a soluble or membrane bound receptor, while nitric oxide (NO), an oxidative stress molecule, diffuses through the cell membrane without a receptor. Both mediators signal through different mechanisms, yet they are dependent on NFκB. We proposed that both mediators are co-induced and co-regulated in inflamed mammary epithelial cells.

**Methods:**

SCp2 mammary epithelial cells were treated with bacterial endotoxin (ET) for different time periods and analyzed for induction of IL-6 secretion and NO production by ELISA and Griess reaction, respectively. The expression of *IL-6 *and *induced NO synthase (iNOS) *was assayed by real time PCR and/or western immunoblots, and the activation of NFκB was assayed by immunobinding assay. To investigate the role of mammary cell microenvironment (cell-substratum or interaction of mammary epithelial cell types; critical to mammary development, function, and disease) in modulation of the inflammatory response, SCp2 cells were cultured with or without extracellular matrix (EHS) or in coculture with their myoepithelial counterpart (SCg6), and assayed for ET-induced IL-6 and NO.

**Results:**

Endotoxin induced NFκB activation at 1 h after ET application. IL-6 secretion and NO production were induced, but with unexpected delay in expression of mRNA for *iNOS *compared to *IL-6*. NFκB/p65 activation was transient but NFκB/p50 activation persisted longer. Selective inhibition of NFκB activation by Wedelolactone reduced ET-induced expression of IL-6 mRNA and protein but not iNOS mRNA or NO production, suggesting differences in IL-6 and iNOS regulation via NFκB. SCp2 cells in coculture with SCg6 but not in presence of EHS dramatically induced IL-6 secretion even in the absence of ET. ET-induced NO production was blunted in SCp2/SCg6 cocultures compared to that in SCp2 alone.

**Conclusions:**

The differential regulation of IL-6 and iNOS together with the differential activation of different NFκB dimers suggest that IL-6 and iNOS are regulated by different NFκB dimers, and differentially regulated by the microenvironment of epithelial cells. The understanding of innate immune responses and inflammation in epithelia and linkage thereof is crucial for understanding the link between chronic inflammation and cancer in epithelial tissues such as the mammary gland.

## Background

Epithelial cells form the first line of contact with pathogens and are capable of initiating and orchestrating the innate immune response against external insults [1]. However, a clear understanding of the regulation of inflammatory respondents and the role of the microenvironment in such regulation are still missing. Mammary epithelial cells, unlike other epithelial cells such as intestinal or skin cells, are well defined for their responsiveness to signals for proliferation (hormone signal) and differentiation (hormone and extracellular matrix signals) in the different stages of development of the mammary gland [2]. However, these epithelial cells are poorly understood for their responses to dedifferentiation signals from inflammatory stimuli such as bacterial endotoxin (ET), and whether inflammatory responses of the mammary epithelium are modulated by developmental stage or cell microenvironment, despite the suggested link of chronic inflammation in epithelia to eventual development of cancer therein [3].

The functional mammary epithelium is comprised of a monolayer of SCp2 secretory epithelial cells open to the alveolar lumen and surrounded by a layer of contractile myoepithelial SCg6 cells [4]. The ratio of SCp2 to SCg6 cells increases across development and differentiation of the mammary gland. SCp2 secretory epithelial cells in culture respond to exogenous extracellular matrix (ECM) or intercellular interactions (co-culture with myoepithelial counterpart SCg6) in the presence of lactogenic hormones, by forming cell clusters and induction of β-casein expression [4,5], thus mimicking the differentiation and normal function of mammary epithelial cells *in vivo *wherein the two cell types organize to form the bilayered secretory epithelium of the mammary gland. SCp2 cells are responsive to ET by activation of the cytosolic transcription factor NFκB, by secretion of inflammatory cytokines such as IL-6 and TNFα, and by reverting to a non-differentiated state depicted by a downregulation of β-casein as well as other differentiation markers [6,7].

The mammalian NFκB family is comprised of five subunits: p65 (RelA), RelB, c-Rel, p50/p105 (NFκB1) and p52/p100 (NFκB2) that combine in different homo and hetero dimers to form active NFκB. NFκB is found inactive in the cytosol due to binding to inhibitory kappa B (IκB). Upon stimulation, IκB kinase (IKK) phosphorylates IκB and labels it for ubiquitin-dependent degradation, thereby releasing activated NFκB which then translocates to the nucleus to activate target genes [8]. Recent studies have suggested NFκB to be the missing link between inflammation and cancer since it plays a critical role not only during inflammation but also in regulating cell cycle, cell differentiation and other normal functions of the cell [9]. Induced inflammation in the absence of immune cells activates NFκB to induce an array of inflammatory respondents such as cyclooxygenase-2 (COX-2), matrix metalloproteinases, inflammatory cytokines (IL-6, TNF-alpha, etc.), and iNOS/NO production.

IL-6 is a multifunctional cytokine produced by immune cells as well as non-immune cells such as endothelial, fibroblast and epithelial cells, and is often a marker of acute or chronic inflammation in clinical diagnostic assays [10] and found to be critical for cell survival and development of certain cancers [11].

Nitric oxide (NO) is a pleiotropic inflammatory marker produced by the conversion of L-arginine to L-citrulline via three different types of nitric oxide synthase (NOS) [12]. Neuronal NOS (nNOS or NOS-1) and endothelial NOS (eNOS or NOS-3) are constitutively expressed in neuronal and endothelial cells, respectively; while the induced NOS (iNOS or NOS-2) is induced by inflammatory stimuli such as endotoxin (ET) and cytokines such as interferon gamma (IFN-γ) and IL-1β in a variety of cell types, including epithelial cells, and produces high concentrations of NO [13]. The function of NO varies from a potent vasodilator and neurotransmitter to inducer of pathogen death and tissue damage depending on its concentration in the tissue [14]. However, the role of NO in epithelial inflammation is poorly defined and subject to multiple interpretations of its causal effects.

In addition to their involvement in the inflammatory response, IL-6 and NO also may affect epithelial cell development and function through cell regulation (IL-6) [15] and intervention in cell signaling (NO) [16], with potential for different effects at different stages of mammary gland development. Therefore, studying the regulation of these inflammatory markers and their common regulator (NFκB) in a differentiation-competent and microenvironment responsive mammary epithelial system allows investigation of the response of specific epithelial cell types to external inflammatory stimuli under different conditions (growth, differentiation, and acute or chronic inflammation) which model those of their parent epithelial tissues, and in the absence of immune cells. The understanding of such innate immune responses of epithelia and the linkage thereof to chronic inflammation is crucial for understanding the link between chronic inflammation and cancer in epithelial tissues.

The focus of this study was to investigate the regulation and coordination of IL-6 and iNOS by ET-induced NFκB activation in mammary epithelial cells, and whether such inflammatory responses are modulated by the cell microenvironment in order to further understand inflammation and inflammation-associated cell transformation in epithelial cells.

## Methods

### Cell lines and materials

Mouse mammary epithelial cells SCp2 and SCg6 were kindly provided by Dr. Pierre Desprez, (Geraldine Brush Cancer Research Institute; San Francisco, CA). Bovine insulin, ovine prolactin, hydrocortisone, endotoxin (ET, as *Salmonella typhosa *lipopolysaccharide >500,000 EU (ET units)/mg), and dimethyl sulfoxide (DMSO) were purchased from Sigma (St. Louis, MO). Englebreth-Holm-Swarm (EHS)-Matrix growth-factor-reduced BD Matrigel™ (a commercially available extracellular matrix, ECM) was purchased from BD Biosciences (Bedford, MA). Complete™ protease inhibitor tablets were purchased from Roche Diagnostics (Mannheim, Germany). Wedelolactone was purchased from EMD Biosciences (La Jolla, CA). Tetramethyl benzidine (TMB) peroxidase substrate was purchased from BioFX Laboratories (Owings Mills, MD). HRP conjugated anti-rabbit and anti-mouse IgG and enhanced chemiluminescence reagent (ECL) were purchased from General Electric (GE) Healthcare (Buckinghamshire, UK).

### Cell culture

Low passage number (13 to 15) SCp2 cells were used throughout. Cells were maintained in growth medium (5% FBS-GM) comprised of DMEM/F12 containing 5% fetal bovine serum (FBS), insulin (5 μg/ml) and gentamicin (50 μg/ml) at 37°C in a humidified atmosphere (95% Air; 5% CO_2_).

### ET-induced inflammation in SCp2 mouse mammary secretory-epithelial cells

To assay for the inflammatory responses of differentiating mammary cells, SCp2 cells were plated on plastic in 5% FBS-GM. Twenty four hours later, cells were induced to differentiate by adding differentiation medium (0% FBS-DM) comprised of DMEM/F12, gentamicin (50 μg/ml), lactogenic hormones (insulin (5 μg/ml), prolactin (3 μg/ml), and hydrocortisone (1 μg/ml)) lacking FBS but supplemented with 0 or 1.5% (v/v) exogenous extracellular matrix (Matrigel™ (EHS)). A stock solution of ET was prepared at 1 mg/ml in 0% FBS-DM. Inflammation was induced by application of a non-toxic dose of ET (10 μg/ml) in 1% FBS-DM 24 h after inducing differentiation. Samples were harvested at 0, 1, 3, 6, 12, 24, and 48 h after ET addition. The collected medium was supplemented with Complete™ protease inhibitor and stored at -80°C for later analysis. Cells were immediately washed and processed for RNA extraction or nuclear and cytosolic protein extraction. To assay for β-casein expression, cultures were left in differentiation medium for 7 days before harvesting the RNA. Reverse transcribed polymerase chain reaction amplification (RT-PCR) was used to assay for β-casein expression in differentiated SCp2 cells using the following primer set: forward (F) = 5'-GTGGCCCTTGCTCTTGCAAG-3'; reverse (R) = 5'-AGTCTGAGGAAAAGCCTGAAC-3' [17].

### SCp2/SCg6 co-culture system

SCg6 cells were seeded on plastic at 4 × 10^4 ^cells/cm^2 ^in 5% FBS-GM for 24 h, then SCp2 cells were seeded on top. The co-cultured cells were shifted to differentiation medium for 24 h before inducing inflammation by addition of ET (10 μg/ml in 1% FBS-DM). Wells of either SCg6 or SCp2 (both at 4 × 10^4 ^cells/cm^2^) alone plated on plastic were used as controls for the SCp2-SCg6 co-culture response to ET treatment.

### Inhibition of NFκB activation by Wedelolactone, a selective inhibitor of IKKα and IKKβ

Wedelolactone (7-Methoxy-5, 11, 12 -trihydroxy-coumestan), the natural anti-inflammatory agent found in herbal medicines from *Eclipta alba*, is a selective and irreversible inhibitor of IKKα and IKKβ kinase activity (IC50 = 10 μM) that inhibits NFκB-mediated gene transcription by blocking the phosphorylation and degradation of IκBα [18], with no effect on the activities of p38 MAPK or Akt (per the provider; EMD Biosciences). Wedelolactone (5 mg/ml in DMSO) was added at 10 μM to the cells in 1% FBS DM for 1 h prior to addition of ET.

### Immunoassay of Interleukin-6

To measure IL-6 secretion in response to ET in SCp2 cells, medium collected at various times post-ET treatment was assayed by enzyme-linked immunosorbent assay (ELISA) for IL-6 (DuoSet kit; R&D Systems Inc, Minneapolis, MN) according to the manufacturer's protocol. Samples were assayed in duplicate and data is represented as the average pg IL-6/ml of three experiments ± standard error of the mean (SEM).

### Griess reaction assay of NO for NOS activity

The analysis of NO was accomplished by the Griess assay that measures nitrite (the stable spontaneous oxidation product of NO) using a Griess Reagent Kit (Molecular Probes, Eugene, OR) according to the manufacturer's protocol. Samples were assayed in duplicate and data is represented as the average concentration of NO_2_^- ^of three experiments ± SEM (μM ± SEM).

### RNA extraction, reverse-transcription and quantitative real time polymerase chain reaction analysis

Total RNA was harvested from cells using Qiagen RNeasy kits (Qiagen, Valencia, CA) according to the manufacturer's protocol. One microgram of total RNA was treated with DNAse I (Promega, Madison, WI) before synthesizing cDNA using the Promega reverse transcription system (Promega). Quantitative real time PCR (qPCR) was performed using Qiagen Hot start SyBR Green PCR master mix (Qiagen, Valencia, CA) for each of IL-6 (NM_031168, F: 5'-GTTCTCTGGGAAATCGTGGA-3', R: 5'-GGAAATTGGGGTAGGAAGGA-3'), iNOS (NM_010927, F: 5'-CCCTTCCGAAGTTTCTGGCAGCAGC-3', R: 5'-GGCTGTCAGAGCCTCGTGGCTTTGG-3') [19], nNOS (NM_008712), and eNOS (NM_008713) target genes and glyceraldehyde-3-phosphate dehydrogenase (GAPDH) (BC094037, F: 5'-ACCACAGTCCATGCCATCAC-3', R: 5'-TCCACCACCCTGTTGCTGTA-3' [20]) as a reference gene. SybrGreen fluorescence of amplified products was quantified with an MJ Research Opticon 2 reader (BioRad, Hercules, CA) relative to an appropriate standard curve from autonomous qPCR assay reactions. Primer pairs were either adopted from the literature or designed using Primer_3 primer design software [21] and synthesized by Operon Biotechnologies Inc (Huntsville, AL), with amplified products therefrom authenticated by sequencing. Each sample was analyzed in triplicate qPCR reactions with the average quantity for each gene of interest from triplicate PCR reactions normalized against the average quantity for the reference gene (GAPDH) from triplicate PCR reactions. The results of qPCR analysis are presented as the average amount of each gene relative to GAPDH ± SEM.

### Intracellular protein isolation

Total proteins were extracted by scraping SCp2 cells in lysis buffer (10 mM Tris-HCl pH 7.5, 150 mM NaCl, 1% v/v Triton X) supplemented immediately before use by addition of 0.5% sodium orthovandate and 40 μl of Complete™ protease inhibitor solution (1 tablet/2 ml deionized water per manufacturer's instructions). Separate nuclear and cytosolic proteins were selectively extracted (Nuclear Extract kit; Active Motif, Carlsbad, CA) according to the manufacturer's protocol.

### Western immunoblot analysis

Total or cytosolic proteins were resolved by SDS-polyacrylamide gel electrophoresis (SDS-PAGE), blotted onto polyvinylidene fluoride (PVDF, General Electric (GE); Buckinghamshire, UK) transfer membrane, and probed for iNOS (Santa Cruz), phospho-eNOS (ser1177 or Thr495) (cell Signaling Technology), IκBα (Abcam), pIκBα (phospho S32+S36) (Abcam), or β-actin (Sigma). Total protein extracts of primary bovine aortic endothelial cells (BAEC) or rat brain lysates (Cell Signaling Technology) were used as positive controls. Densitometric analyses were performed using NIH image J (NIH).

### Immunobinding assay for NFκB activation

NFκB activation in nuclear proteins was determined using the NFκB family Trans-AM NFκB binding assay kit (Active Motif, Carlsbad, CA) according to the manufacturer's protocol. Positive and negative controls were assayed simultaneously to verify response specificity. Samples were assayed in duplicate, with results shown as the average of absorbance (A690) ± standard deviation (SD).

### Immunodot blot cytokine protein array analysis

RayBio™ Mouse Cytokine ArrayI was purchased from RayBiotech, Inc (Norcross, GA). Conditioned medium collected from control and ET-treated SCp2 cells at 1, 3, 6, and 12 h post-ET was incubated with the cytokine array membranes according to RayBiotech protocols. Signal was detected using a provided ECL Plus detection system (Amersham Phramacia Biotech) and exposed to Kodak x-omat AR film (Kodak; New Haven, CT).

### Statistical analysis

Significant differences between different groups were determined using Proc Mixed analysis of SAS 9.1 (SAS Institute Inc., Cary, NC). For each set of experiments studying the effect of ET alone or ET in the presence or absence of Wedelolactone (IKK inhibitor), serum and ECM on IL-6, iNOS/NO and/or NFκB, the statistical analysis included time post ET treatment, treatment (ET, Wedelolactone, ECM, or ET and Wedelolactone), and time by treatment interactions. The effect of treatment within each time point was tested using the slice option by time. Results of two experiments were expressed as mean ± SEM, and significance was defined by p < 0.05, unless noted otherwise.

## Results

### ET induced IL-6 and NO in SCp2 mouse mammary epithelial cells

**Figure 1:**
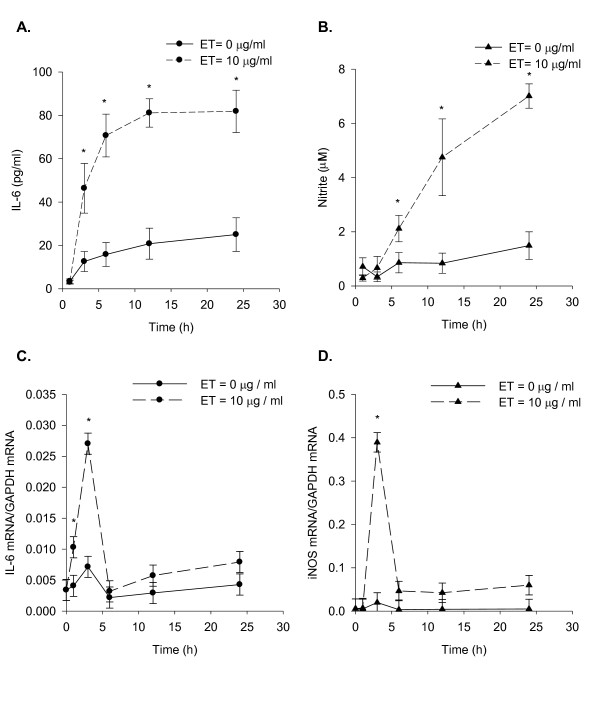
**Temporal pattern of ET-induced IL-6 secretion, NO production, and IL-6 and iNOS mRNA expression**. Subconfluent SCp2 cells were treated with ET (0 or 10 μg/ml) in 1% FBS DM as described in Methods. (A) IL-6 secretion assayed by ELISA and (B) NO production assayed by Griess reaction. RNA harvested at each time point were analyzed by RT-qPCR for ET-induced (C) IL-6 mRNA and (D) iNOS mRNA and quantified relative to GAPDH mRNA for each. Solid-line represents the results for control non ET-treated cells; while dashed-line represents the results for ET-treated cells. Each experiment was performed at least 3 times. Samples were assayed separately in duplicate analyses by either ELISA or Griess reaction assay. For RT-qPCR, each sample was analyzed in triplicate RT-qPCR reactions. Data represents the average of samples from 3 experiments ± SEM with (*) denoting significant differences from non-ET control within each time point (p < 0.05).

To compare the temporal pattern of ET-induced IL-6 secretion and NO production, the medium of ET treated SCp2 cells was analyzed for IL-6 and NO concentrations at sequential time points. IL-6 secretion was significantly increased by 3 h post-ET and continued to increase until it plateaued at 12 h and after (Figure 1A). A slight but not statistically significant increase of IL-6 secreted protein was observed over time even in the absence of ET (Figure 1A). In contrast, nitrite concentrations, reflecting NOS activity, showed no increase until 6 h post-ET treatment but increased continually thereafter for the duration of the experiment (Figure 1B). In the absence of ET, basal nitrite levels changed little over time (Figure 1B). IL-6 mRNA expression was sharply induced as early as 1 h after ET treatment, and peaked at 3 h before decreasing to control concentrations by 6 h post-ET treatment (Figure 1C). A subsequent slow rise in the expression of IL-6 mRNA was also observed with time between 12 and 24 h post-ET in both ET-treated and control cells (Figure 1C). In contrast, iNOS mRNA expression increased sharply from near zero at 1 h post-ET to peak at 3 h post-ET with maximum expression 10-15 fold higher than that for induction of IL-6 mRNA relative to GAPDH at the same time point. By 6 h post-ET, iNOS mRNA expression had decreased sharply and was maintained near control concentrations for the remainder of the experiment (Figure 1D).

### Immunoblot analysis of the protein expression of the three isoforms of NOS and phosphorylation status in mammary cells

**Figure 2:**
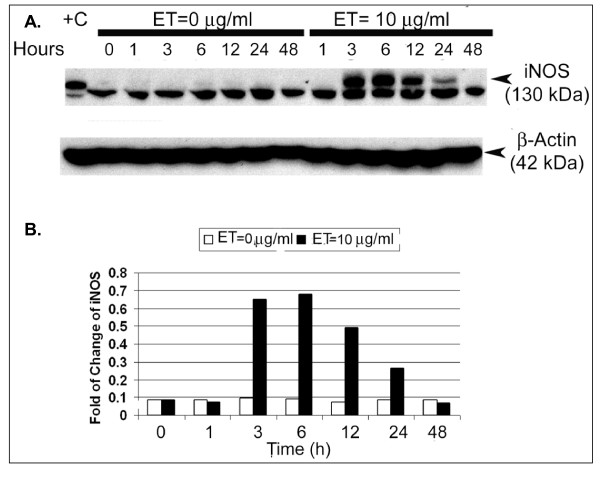
**ET-induced expression of iNOS protein in SCp2 cells**. Western immunoblots of total cellular protein extracts were probed for (A) iNOS with expected molecular weight of 130 kDa (specific bands indicated by arrow). (B) Densitometric analysis was done using NIH Image J program. The fold change of the intensity of the 130 KDa band relative to that of β-actin housekeeping gene expression was plotted over time post-ET treatment. Bovine aortic endothelial cell (BAEC) total protein extract were included as positive control (+C) for iNOS. Equal loading of proteins was verified by probing for 42 kDa β-actin. Each lane represents one sample at a particular time point. This experiment was repeated three independent times and densitometric analysis shown was performed on one of the blots as representative of the magnitude of change noted across the three experiments.

Western immunoblotting analysis showed no expression of iNOS protein in SCp2 cell in the absence of ET; however, a target 130 kDa band appeared maximally at 3 h through 6 h post-ET treatment before declining from 12 through 24 h to disappear by 48 h (Figure 2A &2B). An inexplicable lower, but apparently non-specific unidentified band at ~120 kDa appeared in all samples assayed independent of ET treatment, including the bovine aortic endothelial cells (BAEC) protein extract used as positive control (Figure 2A). Immunoblots for the other two forms of NOS in SCp2 cells showed no detectable nNOS expression (data not shown), and only a basal expression of eNOS which showed no modulation by ET treatment (data not shown) and showed no evidence of activation based on lack of phosphorylation of amino acid residues serine 1177 or threonine 495 except in the BAEC positive control (data not shown).

### ET activation of NFκB subunits p65 & p50 in SCp2 cells

**Figure 3:**
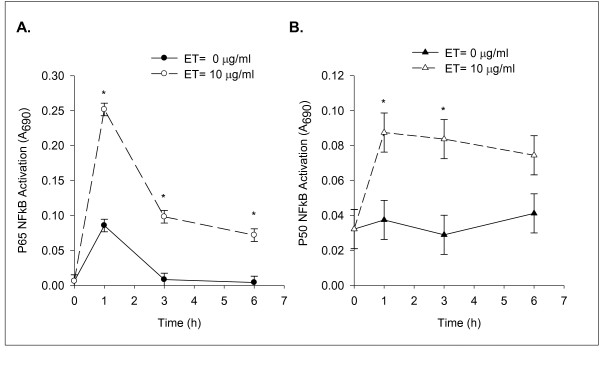
**ET induces binding activity of NFκB p65 and p50 in SCp2 cells**. SCp2 cells were treated as described in Methods with 0 or 10 μg of ET per ml in 1% FBS-DM for 0, 1, 3, and 6 h. Isolated nuclear proteins of control (0 μg ET) and ET-treated (10 μg ET) SCp2 cells were analyzed for (A) NFκB p65 or (B) NFB p50 binding activities by immunobinding assays, with relative binding activity shown as A_690 _above blank. Solid-line and closed symbol represent the results of control non ET-treated samples, while dashed-line and open symbol represent the results of samples from ET-treated cells. This experiment was performed at least twice. Data represents the average for duplicate samples ± SD of a representative experiment. (*) denotes significant difference among treatment within each time point (p < 0.05).

We defined the temporal pattern of NFκB activation by ET, and compared that pattern to the temporal pattern of ET-induced IL-6 and iNOS mRNA expression and NO formation. The nuclear proteins isolated from ET-treated SCp2 cells were analyzed for the different forms of NFκB using the NFκB family binding assay. Of the 5 subunits of NFκB tested, two forms, p65 and p50, increased their binding activity in response to ET compared to the basal binding activity in SCp2 cell nuclear protein extracts. Figure 3A shows an abrupt increase in ET-induced p65 activity that peaked by 1 h post-ET treatment, decreased thereafter to 1/3 and 1/4 the peak level at 3 h and 6 h, respectively, but was well above the level seen in non-ET treated cells at the corresponding time. In contrast, p50 activity was increased to maximum by 1 h post-ET and declined only slightly by 6 h post-ET (Figure 3B).

### Wedelolactone inhibits ET-induced IKK phosphorylation

**Figure 4:**
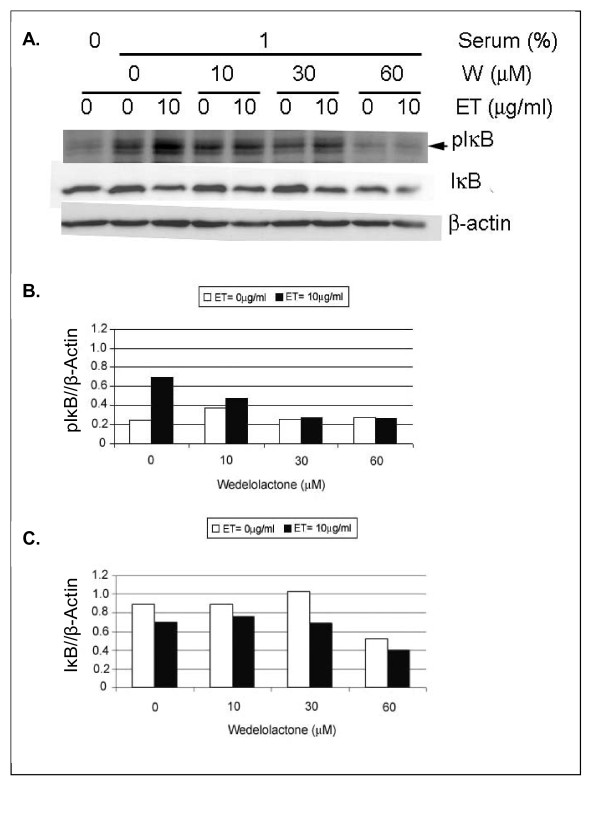
**Wedelolactone inhibits ET-induced IKK phosphorylation**. (A) Nuclear protein of SCp2 cells treated with various doses of the IKKα/IKKβ inhibitor, Wedelolactone (W), were harvested and assayed for phosphorylated and total IκB proteins. Equal loading of proteins was verified by probing for 42 kDa β-actin. (B & C) Densitometric analysis was calculated using NIH Image J program. The intensity of each of the phospho IκB (B) and IκB (C) bands were compared to those of β-actin housekeeping gene expression. Each lane represents one sample at a particular time point. This experiment was repeated two independent times. The same trend of change appeared in both plots, but the blot and densitometric analysis shown represent the stronger change of the two.

SCp2 cells were treated with Wedelolactone for 1 h prior to ET addition, and isolated proteins were assayed by western immunoblots for phosphorylated IκB 1 h post-ET. Phosphorylation of IκB increased 3 fold in response to ET treatment (Figure 4A &4B). However, treatment of SCp2 cells with Wedelolactone prior to ET addition reduced phosphorylated IκB in a concentration dependent manner (Figure 4B). Immunoblots of total IκB showed no clear decrease in IκB protein (Figure 4C) except at the highest Wedelolactone concentration (Figure 4A &4C).

### Wedelolactone inhibits ET-induced IL-6 but not iNOS mRNA expression

**Figure 5:**
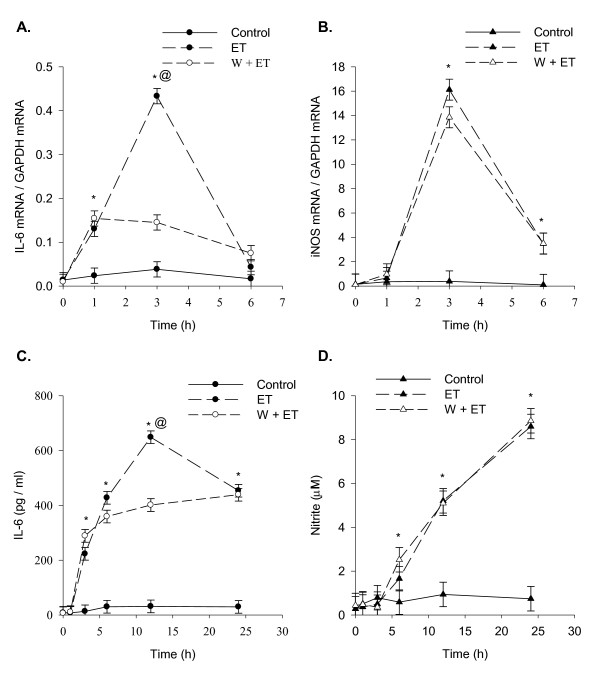
**Wedelolactone inhibits ET-induced IL-6 but not iNOS**. Total RNA was extracted from SCp2 cells treated with 0 or 10 μM Wedelolactone (W) in DMSO (vehicle) for 1 h before ET treatment at 0 or 10 μg ET/ml for 0, 1, 3, and 6 h. 1 μg total RNA were reverse transcribed then amplified by real-time qPCR for ET induced (A) IL-6 and (B) iNOS mRNA. Amplified mRNA concentrations of IL-6 and iNOS were normalized to those of constitutive GAPDH mRNA. The medium was collected at 1, 3, 6, 12, and 24 h post-ET, and assayed for induced (C) IL-6 secretion and (D) NO production. Controls (DMSO only or Wedelolactone only (not shown)) induced basal levels of both IL-6 and NO production. Data represents the average for duplicate samples ± SD of a representative experiment. (*) denotes significant difference between ET and control groups and @ denotes significant difference between W+ET and ET only treated cells within each time point (p < 0.05).

Wedelolactone (10 μM) reduced IL-6 mRNA expression by ~70% (p < 0.05) at 3 h after ET treatment compared to that of cells treated with ET in the absence of Wedelolactone (Figure 5A). However, expression of ET-induced IL-6 mRNA was incompletely inhibited by 10 μM Wedelolactone as shown by its significant elevation above control at 1 and 3 h post-ET (Figure 5A). By 6 h, ET-induced IL-6 mRNA expression had decreased to non-ET control levels (at 0 h) independent of Wedelolactone treatment (Figure 5A). In contrast, Wedelolactone did not inhibit iNOS mRNA expression in SCp2 cells at 3 h post-ET (Figure 5B). By 6 h after ET treatment, iNOS mRNA decreased to near pre-ET control levels but remained significantly higher than control levels (Figure 5B) independent of Wedelolactone pre-treatment.

Despite strong inhibition of IL-6 mRNA expression (~70% inhibition) by Wedelolactone (Figure 5A), ET-induced IL-6 protein secretion showed inhibition of (38% (p < 0.05)) only at 12 h post-ET compared to control cells treated with ET alone in the absence of Wedelolactone (Figure 5C). In contrast, ET-induced NO production was not affected by Wedelolactone pretreatment (Figure 5D), consistent with the lack of effect of Wedelolactone on ET-induced iNOS mRNA expression (Figure 5B).

### Effects of exogenous EHS on ET-induced IL-6 secretion and NO production in SCp2 cells

**Figure 6:**
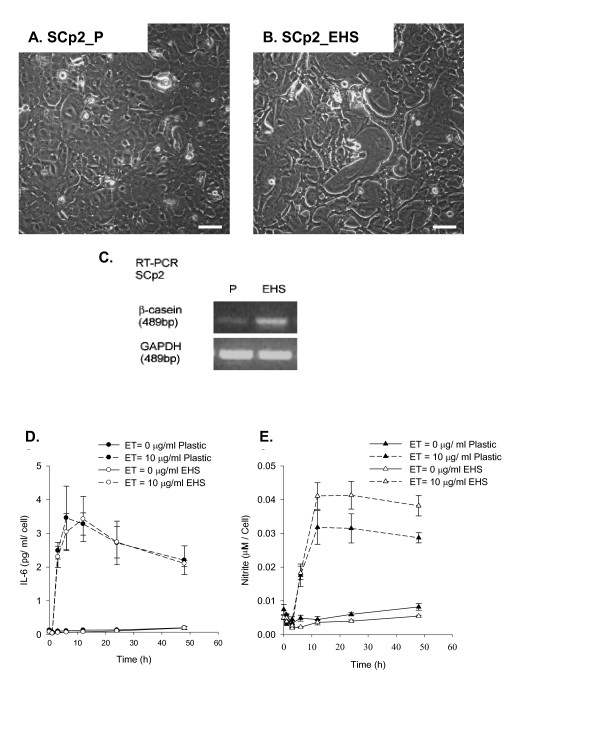
**The effect of EHS on SCp2 cell differentiation and their response to ET**. Phase contrast photomicrographs (40×, Bar = 50 μm) of SCp2 cells (plated at 4 × 10^4 ^cell/cm^2^) on day 3 of culture on (A) plastic (SCp2_P), or (B) in the presence of EHS (SCp2_EHS). (C) Expression of β-casein assayed by RT-PCR. GAPDH PCR product was used as a normalizing control. (D & E) SCp2 cells were plated as described in Methods. The medium was collected and assayed for (D) IL-6 secretion (pg/ml) and (E) nitrite production (μM) normalized to cell number. Open symbols depicts the presence of EHS. Dashed lines depict the ET treatment. Data represents the average for triplicate samples ± SD of a representative experiment.

To confirm that SCp2 cells are differentiation competent as shown by others [4,5], cells were grown under growth (plastic substrate without lactogenic hormones) (Figure 6A) or differentiation conditions (EHS and lactogenic hormones). Differentiated SCp2 cells were marked by their reorganization into cell clusters (Figure 6B) and upregulation of the milk protein β-casein, assayed for by RT-PCR (Figure 6C).

Next, we tested the effect of EHS addition on ET-induced inflammation in mammary secretory epithelial cells. SCp2 cells were grown and treated with ET as described in Materials and Methods. The medium was collected at 0, 1, 3, 6, 12, 24, and 48 h post ET treatment and was assayed for IL-6 secretion (Figure 6D), and NO production (Figure 6E). In order to compensate for any difference in cell growth rate on EHS vs. plastic, the results of secreted IL-6 and NO production were presented relative to cell number. SCp2 cells showed similar temporal patterns and concentrations of IL-6 secretion in response to ET treatment in the absence or presence of EHS (Figure 6D). Similarly, the temporal pattern of ET-induced NO production was not modulated by EHS addition; however, the magnitude of NO production tended to be greater, though not statistically significant, in cells supplemented with EHS (Figure 6E). EHS addition had no effect on ET-induced IL-6 and iNOS mRNA expression (data not shown).

### Effect of mixed SCp2 and SCg6 cells on inflammatory response to ET

**Figure 7:**
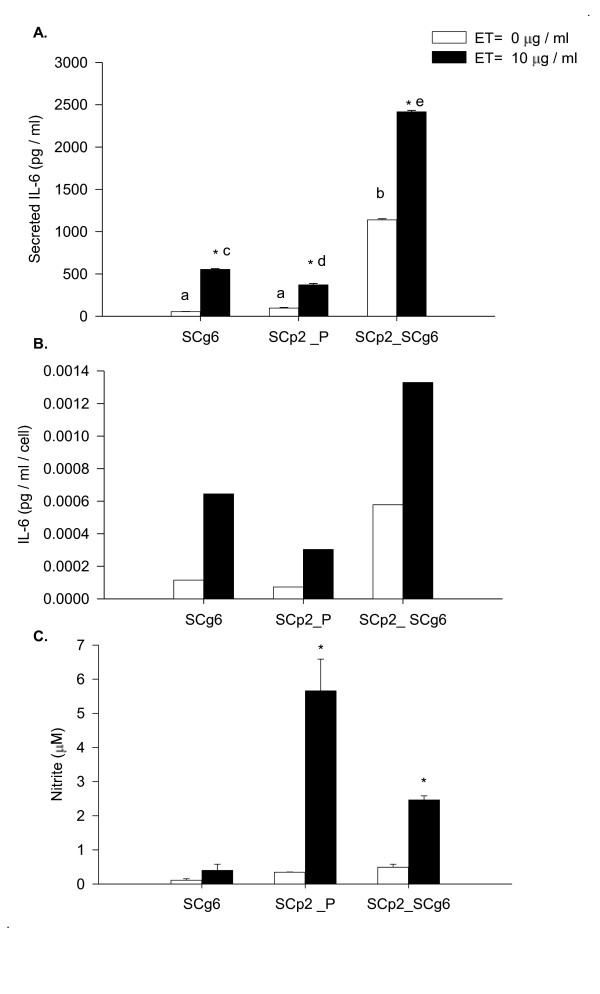
**Effect of SCg6 monolayer on basal and ET-induced IL-6 secretion and NO production in SCp2 cells**. SCg6 and SCp2 cells were cocultured as described in Methods. The medium was collected 24 h later and assayed for basal (open bar) or ET-induced (closed bar) (A) IL-6 secretion and (C) NO production. (B) represents basal and ET-induced IL-6 secretion in SCg6, SCp2 and SCp2:SCg6 coculture normalized to cell number. Data represents the average for IL-6 protein or nitrite concentrations from 2 independent experiments ± SEM. (*) denotes significant differences between treatment within each time point (p < 0.05) and letters denote significant differences among cell types within treatment (p < 0.05).

Both secretory (SCp2) and myoepithelial (SCg6) mammary cell types are important in the formation and differentiation of the bi-layered secretory epithelium in the mammary gland [17,22]. Also, the lactating mammary gland is notably sensitive to microbial ET during intramammary infection [15]. Therefore, we investigated the effect of SCp2 and SCg6 interaction on ET-induced inflammation. Surprisingly, the coculture of SCp2 and SCg6 cells in the absence of ET induced a dramatic increase (p < 0.05) in IL-6 secretion (Figure 7A) that was significantly higher than basal or ET-induced IL-6 secretion in either SCp2 or SCg6 alone (Figure 7A). The concentration of secreted IL-6 remained dramatically higher in medium from SCp2:Scg6 cocultures (Figure 7B) even if normalized to cell number; thus the dramatically increased IL-6 induction in cocultures was not due to higher cell seeding density or growth rate in cocultures vs. individual cell cultures. In contrast, spontaneous NO production was modest in SCp2:SCg6 cocultures (1:1 ratio) in the absence of ET-treatment (Figure 7C). Upon ET treatment, NO production increased but the total concentration was only half the level of NO produced in ET-treated SCp2 cells on plastic (Figure 7C). SCp2 cells alone showed the expected induction of NO by ET, while SCg6 showed little NO production in response to ET (Figure 7C).

### Effect of SCp2:SCg6 cell ratio in coculture vs. SCp2 cell plating density on plastic on ET-induced IL-6 secretion and NO production

**Figure 8:**
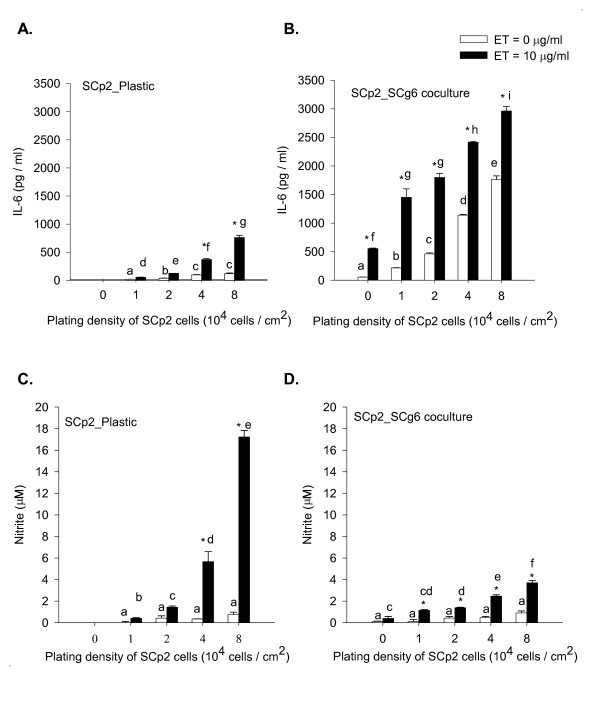
**Effect of SCp2 cell number and substratum on ET-induced IL-6 secretion and NO production**. SCg6 cells were plated and treated as described in Methods. The medium was collected 24 h after ET treatment and assayed for IL-6 secretion (A&B) and nitrite production (C&D) from SCp2 cells (A, C) on plastic or (B, D) on SCg6 monolayer. IL-6 secretion or nitrite production in control cells are depicted by open bars, and those in ET-treated cells are depicted by closed bars. The 0 plating density in (B) and (D) represents data from SCg6 cells alone in the absence of SCp2 cells. Data represents the average of two independent experiments ± SEM. (*) denotes significant differences between ET and non-ET treatment within each time point (p < 0.05) and letters denote significant differences among SCp2 cell plating densities within treatment (p < 0.05).

We studied the effect of ratio of myoepithelial: secretory epithelial mammary cell types in coculture to simulate their estimated ratio in the mammary epithelium across development and functional state. Different cell seeding densities of SCp2 cells (1, 2, 4, and 8 × 10^4 ^cells/cm^2^) were plated either on plastic or on a confluent SCg6 cell monolayer and treated with ET as described in Methods. In the absence of ET, SCp2 cells plated on plastic secreted concentrations of IL-6 that increased modestly with increased cell seeding density (Figure 8A). ET treatment induced a significant 3 to 4 fold increase in IL-6 secretion above basal levels, especially at the two highest SCp2 cell densities (4 × 10^4 ^and 8 × 10^4 ^cell/cm^2^) (Figure 8A). In SCp2:SCg6 cocultures (Figure 8B), the basal IL-6 secretion (without ET) was dramatically higher and increased progressively with increasing plating density of SCp2 cells on a confluent SCg6 monolayer (Figure 8B). A significant (p < 0.05) increase in secreted IL-6 was observed upon treatment with ET for 24 h. However, the 2-3 fold relative increase in ET-induced IL-6 secretion over basal IL-6 secretion in the absence of ET found at lower ratios of SCp2:SCg6 cocultures was decreased at higher SCp2 plating densities. Basal IL-6 secretion was 5 to 9 fold higher (p < 0.05) for SCp2 cells in cocultures (Figure 8B) than for those seeded on plastic (Figure 8A). However, ET induced a 3 to 4-fold higher (p < 0.05) IL-6 secretion in SCp2:SCg6 cell cocultures (Figure 8B) than SCp2 cells on plastic (Figure 8A), regardless of cell plating number. SCg6 alone in the absence of SCp2 showed a 5 fold increase (p < 0.05) in IL-6 secretion in response to ET in comparison to non-ET treated cells (Figure 8B, 0 SCp2 plating density).

In contrast to IL-6 secretion, basal NO production was low in SCp2 cells plated either on plastic (Figure 8C) or in coculture (Figure 8D). ET treatment induced a very significant increase in NO production (p < 0.05) only in SCp2 cells seeded on plastic at 4 × 10^4 ^cell/cm^2 ^(same plating density as previous experiments) or higher (Figure 8C). ET-induced NO production was significant but much lower in SCp2 cells plated on SCg6 monolayer (Figure 8D). Though basal levels of NO were not significantly different in SCp2 plated on plastic or in coculture with SCg6 for all SCp2 cell plating numbers, ET-induced NO levels were significantly (p < 0.05) attenuated in SCp2:SCg6 cocultures (Figure 8D) compared to ET-induced NO levels in SCp2 cells on plastic but only for higher SCp2 plating cell numbers of 4 × 10^4 ^and 8 × 10^4 ^cell/cm^2 ^cell (Figure 8C). In contrast to SCp2 cells, and as previously found (Figure 7B), SCg6 cells alone showed little induction of NO in response to ET (Figure 8D, 0 SCp2 plating density).

### ET induces an array of cytokines in SCp2 mouse mammary cells

**Figure 9:**
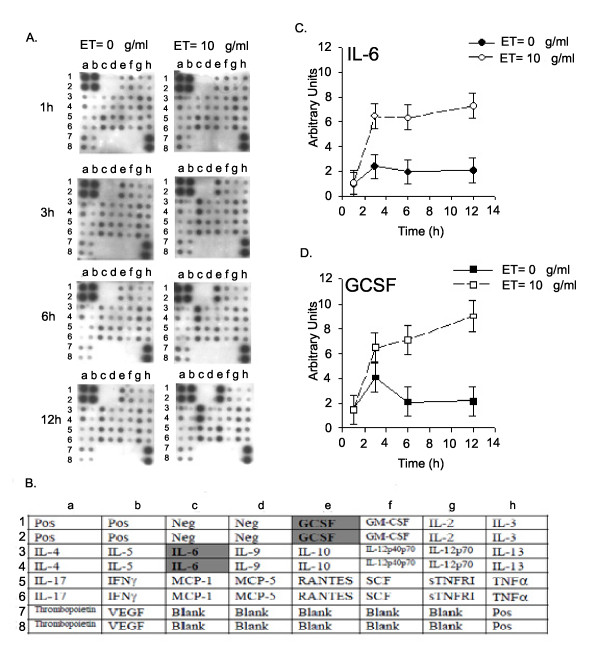
**Mouse cytokine protein array of SCp2 cells following treatment with ET**. Subconfluent SCp2 cells were treated with ET (0 or 10 μg/ml) in 1% FBS DM for 1, 3, 6, and 12 h, and medium collected at each time point was assayed for different inflammatory cytokines (A) by the RayBio Mouse Cytokine Antibody Array I. (B) Layout of the mouse cytokine protein array on the membrane. Abbreviations POS = positive control, NEG = negative control. GCSF = granulocyte colony stimulating factor, GM-CSF = granulocyte macrophage colony stimulating factor, IL = interleukin, IFN-γ = interferon gamma, MCP = monocyte chemotactic protein, RANTES = regulated on activation, normal T expressed and secreted, SCF = stem cell factor, sTNFR = soluble tumor necrosis factor α receptor, TNFα = tumor necrosis factor alpha, VEGF-vascular endothelial growth factor. Densitometric analysis of (C) IL-6 and (D) GCSF proteins secreted in response to ET at different time points was calculated using NIH Image J program. Error bars denote the standard deviation (SD) between duplicate of a representative dot blot.

Culture medium collected from SCp2 cells at 1, 3, 6 and 12 h after ET treatment was analyzed for inflammatory cytokine secretion using RayBio Mouse cytokine Antibody Array I (Fig, 9A &9B). Of the 22 cytokines assayed, only IL-6 (Figure 9C) and granulocyte colony stimulating factor (GCSF) (Figure 9D) increased significantly starting at 3 h post-ET treatment and remained elevated up to 12 h post-ET. Though RANTES appeared to increase slowly starting at 6 h and peaked at 12 h post-ET (Figure 9A) the differences were not statistically significant.

## Discussion

Epithelial cells are important in initiating an innate immune response against external pathogens in tissues such as lung, intestine and mammary gland [1]. Receptors to external pathogens, NFκB activation [1], and cytokine secretion together with production of reactive oxygen and nitrogen species production are critical for local elimination of the pathogen independent of immune cells as well as for activation and recruitment of immune cells to the site of infection [23].

This study investigated the co-regulation of IL-6 secretion and NO production by ET in SCp2 mouse mammary secretory epithelial cell cultures, with prediction of their co-induction by ET as known for immune cells [24]. However, contrary to expectation, they showed different temporal patterns of response to ET in SCp2 mouse mammary secretory epithelial cells. The delayed response of NO relative to IL-6 was observed for both the secreted proteins and their mRNA expression (Figure 1). Although the observed delay in NO production might be partly explained by the time required for iNOS to synthesize NO and the rate of the conversion of the latter to a more stable nitrite (NO_2_^-^) form, the observed delay in expression of iNOS mRNA which was not induced before 3 h post-ET (Figure 1D) compared to that of IL-6 mRNA induced at 1 h post-ET (Figure 1C), indicates differences in the regulation of expression of IL-6 and iNOS as ET-induced respondents.

Treatment of SCp2 cells with different doses of exogenous mouse recombinant IL-6 protein at doses (0.1, 1, and 10 nM) representing respectively 1/10, 1, and 10 × the levels of secreted IL-6 protein found in the medium of ET-treated SCp2 cells, did not induce expression of iNOS when applied alone in the absence of ET (data not shown), suggesting that the expression of iNOS mRNA was induced in response to ET alone and not in response to IL-6, and thus the delay in iNOS expression is due to a different control mechanism than that of IL-6.

We also investigated the effect of ET on the binding activity of p65 and p50, the major NFκB subunit types known to be involved in ET signaling via the rapid MyD88-dependent pathway (reviewed in [25]). The results in Figure 4 showed a rapid activation of both p65 and p50 at 1 h post-ET; however, while p65 activation was transient, activation of p50 was sustained through 6 h post-ET. Although others described the activation of p65/p50 NFκB via the rapid MyD88-dependent pathway involving IKK-dependent IκB phosphorylation and ubiquitin-dependent degradation (reviewed in [25]), other reports suggested that the regulation of p50 might involve additional pathways other than IκB degradation [26]. In any case, the difference in p65 and p50 activation suggests that the difference between ET-induced IL-6 and iNOS mRNA expression might involve different pathways of NFκB activation; one that is IκB degradation-dependent while the other is not; therefore, inhibition of the IKK complex that phosphorylates IκB and labels it for degradation would modulate only the IκB dependent NFκB activation pathway and its target genes.

Consistent with the difference in regulation of both IL-6 and iNOS mRNA expression, Wedelolactone treatment inhibited only ET-induced IL-6 mRNA expression but not that of iNOS. These results suggest that while IL-6 mRNA expression is likely dependent on the rapid MyD88-dependent NFκB activation pathway, iNOS mRNA expression likely involves the delayed MyD88-independent NFκB activation pathway. Kawai et al (2000) showed that ET-induced IL-6 production was impaired in MyD88^-/- ^mouse macrophages [27], while Schilling et al (2002) reported that expression of iNOS but not that of IL-6 mRNA was enhanced by ET-induced IFNγ in mouse macrophages [28]. In our study, IFNγ was not significantly induced in SCp2 cells treated with ET (Figure 9). Hence, these studies, together with our results in epithelial cells suggest that the differential regulation of ET-induced IL-6 and iNOS by NFκB involves different kinetics and alternate pathways of NFκB activation for each whereby IL-6 mRNA expression is likely induced by the rapid MyD88-dependent activation pathway of NFκB but iNOS mRNA expression might involve the slower MyD88-independent activation pathway of NFκB. Furthermore, the observed delay in expression of iNOS compared to that of IL-6 mRNA coincides with reports describing a role for IL-6 in acute inflammation and in the transition to chronic inflammation [10], in contrast to NO that was proposed to limit inflammation [29].

Though iNOS is the induced form of NOS upregulated in response to ET in the SCp2 secretory mammary cells used in this study, other studies described a possibility for induction of mRNA expression of supposedly constitutive eNOS and nNOS or the regulation of eNOS by phosphorylation [12]. Others also showed that the constitutive activity of eNOS can be modulated by phosphorylation of amino acid residues serine 1177 (Ser 1177) and/or threonine 495 (Thr 495) in rat heart and endothelial cells [30]. In this study, we were unable to show conclusive qPCR amplification or modulation of protein expression of either eNOS or nNOS in response to ET (data not shown). Thus, our data suggest that ET-induced NO in SCp2 mammary epithelial cells is due mainly to iNOS expression and activity.

Moreover, by global immunodot-blot assay for ET-induced cytokines, we found that SCp2 mammary secretory epithelial cells are capable of producing inflammatory cytokines and chemokines similar to immune cells. The cytokines that were significantly upregulated in response to ET from immunoblot analysis were GCSF and IL-6 (Figure 9). GCSF is involved in monocyte chemotaxis as well as cell differentiation, suggesting that mammary secretory epithelial cells, likely to be the first cells in contact with infecting bacteria of the mammary gland, play an important role in innate immunity and in recruiting immune cells and orchestrating their function. IFNγ was present but was not significantly induced in ET-treated SCp2 cells.

The present study also demonstrates that ET-induced IL-6 secretion and NO production are differentially regulated by the microenvironment of SCp2 cells in culture. Upon induction of cell differentiation either by addition of EHS or by SCp2:SCg6 co-culture, we investigated the role of EHS and SCp2-SCg6 interaction on IL-6 secretion and NO production in SCp2 cells alone or in SCg6 co-cultures with or without ET-induced inflammation. While EHS had no effect on ET-induced IL-6 secretion (Figure 6D), SCp2 cells cultured in the presence of EHS showed a slight but significant increase (~25%) in NO production in response to ET compared to SCp2 cells on plastic (Figure 6E). Despite differential regulation of the secreted products, ET-induced iNOS mRNA had similar temporal patterns of expression regardless of substratum (data not shown). Whether the observed increase in NO in the medium was due in part to increased chemical stability of NO in the presence of EHS was not investigated. Similarly, IL-6 mRNA expression patterns in response to ET were not affected by EHS (data not shown).

However, there was a strong and unexpected spontaneous induction of IL-6 when SCp2 cells were cultured on a monolayer of SCg6 cells; IL-6 concentrations increased dramatically to several-fold higher than for either SCp2 or SCg6 cells alone, independent of ET treatment and cell number (Figure 7A &7B). The concentration of IL-6 in control co-culture cells was higher than the sum of IL-6 secreted in both control SCp2 and SCg6 cells cultured independently, showing a strong synergistic effect of SCg6-SCp2 interaction on IL-6 secretion even in the absence of ET (Figure 7A &7B). These results are interesting in light of reports in the literature of a role for IL-6 in cell-cell association [31]. In marked contrast, growth of SCp2 cells on an SCg6 monolayer seemed to reduce ET-induced NO response although NO basal levels were not affected (Figure 7C), confirming that the previously shown differential regulation of IL-6 and NO extends to SCp2 cells in co-culture. Despite the intriguing effect of SCp2:SCg6 co-culture on spontaneous and induced inflammation, any inference as to how the effects of SCp2:SCg6 interaction would relate to the mammary gland *in vivo *requires caution since SCg6 cells were described as a malignant mammary cell line having lost their responsiveness to ECM regulation and were shown to induce tumors when injected into athymic nude mice [32]. Whether SCg6 cells result in the tumor or induce transformation in epithelial cells is not yet clear; however, the induction of a spontaneous inflammatory response in SCp2 cocultured with SCg6 may be related to the predicate of chronic inflammation leading to cancer in epithelial tissues [3]. Our findings become even more interesting in light of studies by Talhouk et al (2008) and Gudjonsson et al (2002). The first study underlines the importance of heterocellular interactions between SCp2 and SCg6 cells in enhancing expression of connexins and of catenin cell protein interactions as expected for more complete SCp2 cell and epithelial differentiation in vitro [22], while the second emphasizes the importance of myoepithelial cells in maintaining epithelial cell polarity and the bilayered structure of epithelial cells and myoepithelial cells in the normal mammary tissue [33].

The ratio of secretory epithelial to myoepithelial cells varies in ductular vs. alveolar mammary epithelia [34], with the ratio of secretory epithelial to myoepithelial cells low in duct tissue and higher (>1) in the alveolus with dominance therein by the monolayer of secretory epithelial cells surrounded by a discontinuous layer of myoepithelial cells [34]. Therefore, we varied the ratio of SCp2 to SCg6 cells in the co-culture by plating SCp2 cells on a monolayer of SCg6 cells at SCp2:SCg6 cell plating ratios of 1:4, 1:2, 1:1, and 2:1. SCp2:SCg6 co-culture induced a dramatic 5 to 9-fold increase in basal levels of IL-6 secretion in control non-ET treated cells compared to that in SCp2 or SCg6 cells alone on plastic (Figure 8A vs. 8B). The net increase of IL-6 secretion from ET-induction was independent of cell number in SCp2:SCg6 co-cultures, indicating that cell-cell interaction affected only basal secretion of IL-6 proteins but not that induced by ET (Figure 8B). In contrast to IL-6, while basal NO levels varied little in SCp2 cells on plastic (Figure 8C), ET-induced NO production increased significantly (by 15 to 20-fold) only at high SCp2 cell plating density (4 and 8 × 10^4 ^cell/cm^2^) (Figure 8D) compared to basal non-ET treated levels; emphasizing again the differential regulation of IL-6 and NO in mammary epithelial cells. It remains intriguing but unknown whether or how the cellular ratios and influence thereof on regulation of the inflammatory responses in mammary epithelial cells as shown here might relate to the *in vivo *ratios and associations of secretory epithelial to myoepithelial cells within the quiescent adult human mammary gland and its elevated risk of developing breast cancer [35,36].

## Conclusions

In conclusion, we have shown that ET induces IL-6 and iNOS but not eNOS or nNOS mRNA expression in SCp2 mammary epithelial cells. ET-induced IL-6 and iNOS mRNA expression occurs likely via different regulatory mechanisms as shown by a delay in the temporal pattern of ET-induced iNOS mRNA expression and NO production compared to that of IL-6 mRNA expression and protein secretion. The inhibition of ET-induced IL-6 but not iNOS mRNA expression by the IKK inhibitor Wedelolactone suggests that the different regulation of IL-6 and iNOS by ET likely involves rapid (IL-6) vs. slow (iNOS) NFκB activation pathways. Moreover, cell-ECM and cell-cell interactions between the two resident cell types of the mammary epithelium differentially modulate ET-induced IL-6 and NO inflammatory responses in the absence of immune cells. Intriguingly, cell-cell interaction alone induced remarkable secretion of IL-6 but not NO production. These results suggest that the microenvironment context of the inflamed epithelial cell is important for understanding the regulation of inflammation and the link between inflammation and cancer.

Moreover, our study along with previous studies from our group using similar approaches in the mammary epithelial cell system, emphasize that inflammation in mammary epithelial cells would involve a set of early inflammatory respondents (IL-6, TNFα, and NGF [37] and unpublished data), and late respondents (iNOS, NO, and MMPs [37,38]) induced in temporal sequences that may ultimately lead to disruption of cell-substratum interactions and tissue function to eventually lead to disease. Though all of these inflammatory markers are regulated by NFκB, the studies recorded here show that they likely are not regulated by the same NFκB subunits, or NFκB activating machinery. Therefore, understanding of the innate immune response of epithelia particularly for its regulation and coordination, and linkage thereof to immune responses will be crucial for understanding the link between chronic inflammation and cancer in epithelial tissues.

The difference in the regulation of two NFκB mediated inflammatory respondents (IL-6 and NO) described in this study in addition to the temporal regulation of NFκB for specific responses [39] and for NFκB activation by different inflammatory stimuli [40] demonstrates the importance of timing in orchestrating the activation of inflammatory respondents and regulation of inflammation. The ability of SCp2 mammary secretory epithelial cells to respond to inflammatory stimulation as well as to changes in their extracellular matrix environment and interactions between the two cell types comprising the mammary epithelium provides an interesting model to further investigate the difference in temporal regulation of inflammatory respondents and their modulation by the cell differentiation state and by interaction of epithelial cell types in the absence of immune cells.

## Competing interests

The authors declare that they have no competing interests.

## Authors' contributions

SWM and FLS designed the experiments of this study, wrote the manuscript and assembled the figures. SWM conducted all the experiments. RST provided cells, protocols, and critical reviews for this manuscript. All authors read and approved the final manuscript.
